# Straw Return and Nitrogen Fertilization to Maize Regulate Soil Properties, Microbial Community, and Enzyme Activities Under a Dual Cropping System

**DOI:** 10.3389/fmicb.2022.823963

**Published:** 2022-03-15

**Authors:** Li Yang, Ihsan Muhammad, Yu Xin Chi, Dan Wang, Xun Bo Zhou

**Affiliations:** ^1^Guangxi Colleges and Universities Key Laboratory of Crop Cultivation and Tillage, Agricultural College, Guangxi University, Nanning, China; ^2^The Key Laboratory of Germplasm Improvement and Cultivation in Cold Regions, College of Agronomy, Heilongjiang Bayi Agricultural University, Daqing, China; ^3^College of Horticulture and Landscape, Tianjin Agricultural University, Tianjin, China

**Keywords:** straw return, nitrogen fertilization, soil enzymes, soil microbes, soil properties

## Abstract

Soil sustainability is based on soil microbial communities’ abundance and composition. Straw returning (SR) and nitrogen (N) fertilization influence soil fertility, enzyme activities, and the soil microbial community and structure. However, it remains unclear due to heterogeneous composition and varying decomposition rates of added straw. Therefore, the current study aimed to determine the effect of SR and N fertilizer application on soil organic carbon (SOC), total nitrogen (TN), urease (S-UE) activity, sucrase (S-SC) activity, cellulose (S-CL) activity, and bacterial, fungal, and nematode community composition from March to December 2020 at Guangxi University, China. Treatments included two planting patterns, that is, SR and traditional planting (TP) and six N fertilizer with 0, 100, 150, 200, 250, and 300 kg N ha^–1^. Straw returning significantly increased soil fertility, enzymatic activities, community diversity, and composition of bacterial and fungal communities compared to TP. Nitrogen fertilizer application increased soil fertility and enzymes and decreased the richness of bacterial and fungal communities. In SR added plots, the dominated bacterial phyla were Proteobacteria, Acidobacterioia, Nitrospirae, Chloroflexi, and Actinobacteriota; whereas fungal phyla were Ascomycota and Mortierellomycota and nematode genera were *Pratylenchus* and *Acrobeloides*. Co-occurrence network and redundancy analysis (RDA) showed that TN, SOC, and S-SC were closely correlated with bacterial community composition. It was concluded that the continuous SR and N fertilizer improved soil fertility and improved soil bacterial, fungal, and nematode community composition.

## Introduction

Carbon sequestration and long-term sustainability can be improved with residues return to the field (Su et al., 2020a; Lu et al., 2021). Straw is mainly composed of carbon (C), nitrogen (N), and organic matter (OM) (Fan and Wu, 2020), which can effectively enhance soil fertility and mitigate the negative impacts of excessive synthetic fertilizers uses (Zhao et al., 2019; Wang L. et al., 2021). Numerous studies have demonstrated that SR reduced mineral fertilizer application by enhancing nutrient efficiency and improved organic C inputs (Wu et al., 2020; Wang G. et al., 2021). Furthermore, SR has tremendous potential to improve soil health and micro-ecological environment (Su et al., 2020a). Straw decomposition is a complex process (Zhao and Zhang, 2018; Zhao et al., 2019), which is predominantly mediated by soil microorganisms with specialized functions (Zhao and Zhang, 2018). A variety of microbial communities play significant roles in the crop residues decomposition, such as bacteria preferring to decompose labile compounds and dominating straw degradation at the initial stage of decomposition (Mwafulirwa et al., 2021; Wu et al., 2021). In contrast, fungi decompose more abrasive materials principally in the final stages of decomposition (Marschner et al., 2011). The energy and C derived from the crop residues incorporated into the soil are distributed throughout the trophic levels, affecting different soil microorganisms such as soil bacteria, fungi, and nematodes (Chen et al., 2021).

In addition to a biotic component, the abiotic variables such as temperature and soil moisture, soil N, pH, and soil organic carbon (SOC) also affect the decomposition process and soil microbial activity, structure, and community (Geisseler and Scow, 2014; Kamble and Bååth, 2016). Guangxi is located south of China, which is a typical subtropical monsoon humid region and committed to a double-cropping system, where maize (*Zea mays* L.) is one of the main food crops (Huang et al., 2021). Straw decomposition and nutrient release are accelerated by the high temperature in this region (Ren et al., 2020). Residues incorporation into the soil decomposed more rapidly, which releases a variety of mineral nutrients that may be easily available and absorbed by plants (Muhammad et al., 2021). The straw C/N ratio is a factor that determines the rate of decomposition of the material, with lower C/N ratios favoring bacteria while higher fungi ratios can degrade more complex organic molecules such as lignin.

The activity of soil enzymes has long been considered as a fundamental indication of soil quality (Borase et al., 2020). Soil enzymatic activities are generated and released by soil microbes, which are responsible for organic matter degradation (Burns et al., 2013). The activity of soil enzymes can provide insight into the processes of microbial sensitivity to added C and N (Zhao et al., 2016; Muhammad et al., 2022). The addition of C and N to soil and field management approaches may affect different enzymes in different ways (Gong et al., 2021; Huang et al., 2021). Soil sucrase activity can help plants and soil bacteria use sucrose as an energy source by hydrolyzing it into glucose and fructose (Yu et al., 2018; Wu et al., 2020). Similarly, cellulose (S-CL) activity is significantly correlated with sucrase activity when organic matter was added to the soil, showing that the individual substrate can affect an enzyme-catalyzed biological reaction (Salazar et al., 2011). Thus, understanding the relationship between the impact of SR and N application on soil microorganisms and the production of soil enzyme activities is crucial and will provide insight into the fundamental mechanics of SOC changes under long-term SR.

Therefore, this study was conducted to investigate the effects of SR on the soil bacteria, fungi, and nematode communities, enzyme activities, and soil fertility. High-throughput DNA sequencing of PCR-amplified marker genes sequencing technology has recently provided significant insights into the diversity of the microbial community in various fertilizer management practices and SR decomposition (Zhao et al., 2016; Yuan et al., 2018). We conducted a field experiment in a subtropical region of China with two planting patterns (SR and TP) under N fertilizer application. The objectives of the study were (i) to investigate the impacts of N fertilization and SR on the soil microbial diversity and community composition; (ii) to determine the response of soil enzyme activity under various N levels and SR systems; and (iii) to understand how soil microbes and enzyme activities alter SOC and total N under SR and N application.

## Materials and Methods

### Experimental Site

A field experiment was conducted at Agronomy Research Farm of Guangxi University, Nanning, Guangxi, China (22°50’N, 108°17′E) from March to December 2020. The region belongs to a sub-tropical monsoon climate, with a mean annual temperature of 21.7°C and a mean annual precipitation of 1,298 mm. The soil of the experimental site was classified as clay loam, with initial properties of 14.6 g kg^–1^ SOC, 0.8 g kg^–1^ total N, 42.7 mg kg^–1^ available P, 88.5 mg kg^–1^ available K, and pH 6.5 when sampling at 0–20 cm depth.

### Experimental Design

The experiment was conducted in randomized complete block design in split-plot arrangement with three replications. The planting pattern was the main plot and N fertilization was the split-plot factor. The two planting patterns were straw return (SR; spring and autumn maize residue were mechanically crushed stalks in to 2–3 cm and mixed with soil in top 0–20 cm with rotary tillage) and traditional planting (TP; maize straw was removed from the field after harvested) since 2018. The six N fertilizer treatments were control (N0), 100 kg N ha^–1^ (N100), 150 kg N ha^–1^ (N150), 200 kg N ha^–1^ (N200), 250 kg N ha^–1^ (N250), and 300 kg N ha^–1^ (N300).

In 2020, the maize cultivar “Zhengda-619” was sown twice, the first time on March 11th and harvested on July 9th for spring maize, and the second time on August 2nd and harvested on November 30th for autumn maize. The plot size was 4.2 m × 4.2 m, with a planting density of 55,556 plants ha^–1^, having 60 cm row to row space and 30 cm planting space. The recommended basal doses of phosphate fertilizer (calcium magnesium phosphate, P_2_O_5_ content of 18%) and potash fertilizer (KCl, K_2_O content of 60%) were incorporated at a rate of 100 kg ha^–1^ into the soil before sowing. Two-thirds of the N from urea was applied prior to sowing, with the remaining one-third applied during the large trumpet period. Other field management measures were consistent with typical farming procedures.

### Soil Sampling

Five random soil samples from 0 to 20 cm soil depth were obtained from each sub-plot at the jointing stage (V6) and mature stage (R6) before maize harvest. These soil samples were thoroughly mixed, sieved through a 2 mm mesh, and then divided into two parts, one of which was immediately stored at −80°C in the laboratory for molecular analysis. Additionally, the other portion of the soil samples was air-dried at room temperature and then sieved through 0.069 mm mesh for the analysis of enzyme activities, and/or sieved through 0.15 mm mesh for TN and SOC determination.

### Soil Fertility and Enzyme Activities Analysis

Potassium dichromate volumetric and external heating techniques were used to quantify SOC (Chen et al., 2021), while soil TN was measured by the semi–micro kelvin method (Wang G. et al., 2021). The activities of soil enzymes, soil urease (S-UE), soil sucrase (S-SC), and soil cellulase (S-CL) were evaluated using Solarbio analytical kits BC0125, BC0245, and BC0155S, respectively (Science & Technology Co., Ltd., Beijing, China), as per the procedure of the manufacturer.

### DNA Preparation, PCR Amplification, and High-Throughput Sequencing

Total genomic DNA was extracted through using the E.Z.N.A.^®^ soil DNA Kit according to the manufacturer’s instructions (Omega Bio-tek Inc., Norcross, GA, United States). The final DNA concentration and purity were determined using a Nano Drop 2000 UV-vis spectrophotometer (Thermo Scientific, Wilmington, United States), and the DNA quality was determined using 1% agarose gel electrophoresis. A Thermocycler PCR system was used to perform PCR amplification (Gene Amp 9700, ABI, United States). Bacterial primers 338F 5′-ACTCCTACGGGAGGCAGCAG-3′ and 806R 5′- GGACTACHVGGGTWTCTAAT-3′ were used to amplify the V3-V4 hypervariable sections of the 16S rRNA gene (Chen et al., 2018). The barcode primers ITS1F (5′-CTTGGTCATTTAGAGGAAGTAA-3′) and ITS2R (5′-GCTGCGTTCTTCATCGATGC-3′) were used to amplify the fungal rRNA gene in the ITS1 sequence region (Dang et al., 2020). The fungal ITS1 sequence region was used for nematode DNA gene amplification using the barcode primers NF1 5′-GGTGGTGCATGGCCGTTCTTAGTT-3′ and 18Sr2bR 5′-TACAAAGGGCAGGGACGTAAT-3′ (Xue et al., 2019). The PCR products from bacteria, fungi, and nematodes were extracted from a 2% agarose gel, purified using the AxyPrep DNA Gel Extraction Kit (Axygen Biosciences, Union City, CA, United States), and quantified using a QuantusTM Fluorometer (Promega, United States).

### Processing of the Sequencing Data

All three PCR products (bacterial, fungal, and nematode) were purified, pooled in equimolar amounts and paired-end sequenced (2 × 300) on an Illumina MiSeq platform (Illumina, San Diego, CA, United States) by Majorbio Bio-Pharm Technology Co., Ltd. (Shanghai, China) (Zeng et al., 2020). UPARSE version 7.1 was used to cluster the processed sequences into operational taxonomic units (OTUs) that had at least 97% similarity (Gdanetz et al., 2017). The RDP Classifier method was used to assess the taxonomy of the bacterial sequences against the SILVA database (version 128/16S-bacteria database) and fungal against the United States database (version 7.00; fungal-database) with a confidence level of 70% (Zheng et al., 2020). The taxonomy of nematode sequences was analyzed by the RDP Classifier algorithm against the NCBI database (version NT/its-nematode database) using a confidence threshold of 70% (Xue et al., 2019).

### Alpha and Beta Diversity Analysis

An OTU-based analytical technique was used to assess the bacterial, fungal, and nematode diversity in each sample. The OTU richness and diversity of each sample were assessed using QIIME software version v1.8.0, with a sequencing depth of 3% to measure the diversity index and species richness (alpha diversity).

Beta diversity analysis was used across all samples to estimate the community structure comparison index. The beta diversity of genotypes was calculated at the OTU level using weighted UniFrac distances and visualized using main coordinates analysis (PCoA). The QIIME tool was used to group and evaluate the weighted UniFrac distance matrices. They discovered evolutionary connections between various groups and the quantity of those samples.

### Statistical Analyses

The results of enzymatic activities and nutrient contents were analyzed using two-way ANOVA under two planting patterns and six nitrogen fertilizer application rates. The least significant difference (LSD) was used to separate means and interactions, and statistical significance was evaluated at *P* ≤ 0.05. The alpha diversity was calculated utilizing the Chao1 and Shannon diversity indices. Soil nutrient, enzyme activity, and alpha diversity were correlated using R package “pheatmap” (version 3.3.1). Beta diversity was estimated using the Bray-Curtis distance matrix and PCoA. Redundancy analysis (RDA) was used to examine the relationship between soil sample distribution and soil properties using R 4.0 package.^1^ These analyses were carried out to assess community compositions across all samples using OTU composition and to depict the link between bacterial, fungal, and nematode communities and soil properties.

## Results

### Soil Fertility and Enzyme Activities

**FIGURE 1:**
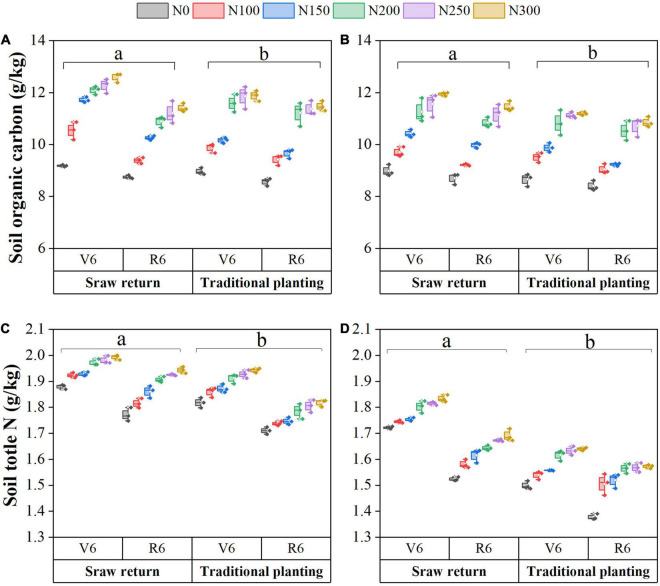
Changes in soil physicochemical properties with straw returning and nitrogen fertilization under dual-cropping system. Soil organic carbon during spring **(A)** and autumn **(B)**, soil total nitrogen content in spring **(C)** and autumn **(D)**.

Straw return had enhanced the soil SOC and TN under both spring and autumn maize cultivation (Figure 1). Compared to the TP treatment, SR significantly increased soil SOC content by 2.8–9.0% and TN content by 6.0–7.1% in both spring and autumn maize cultivated fields (*P* < 0.05). Averaged across SR, SOC and TN contents were 10.7 and 1.9 g kg^–1^ in a spring maize field, and 10.2 and 1.6 g kg^–1^ in an autumn maize field, respectively. Our results showed that SOC contents significantly increased by 7.40 and 2.98% at the V6 stage in spring and autumn maize fields, respectively (Figures 1A,B). In contrast, TN contents decreased by 5.13% in a spring maize field and 6.6% in an autumn maize field (Figures 1C,D). In both spring and autumn maize fields, the SOC and TN contents generally increased with N fertilizer applications. The average SOC and TN contents of N100, N150, N200, N250, and N300 significantly increased compared to the N0 treatment (*P* < 0.05). These results demonstrated 8.8–32.1% changes in SOC and 3.1–8.5% changes in soil TN; however, no significant differences were observed between N200 and N250, and N250 and N300 treatments (*P* > 0.05).

The combined application of straw and nitrogen fertilizer (Table 1) improved soil enzyme activities for both seasons (S). Averaged across two stages, the SR increased S-UE, S-SC, and S-CL by 5.5, 3.5, and 5.8% in spring and 5.9, 18.4, and 12.2% in autumn, respectively (Table 1). Nitrogen fertilization significantly increased the soil enzyme activities in both planting patterns and seasons, suggesting that the S-UE, S-SC, and S-CL activities were significantly boosted in N300 compared to N0 treatment. However, the N250 treatment was not statistically different from N200 and N300, respectively. On an average basis, S-UE, S-SC, and S-CL activities were 290.1, 32.7, and 8.99 U g^–1^ in spring, and 216.3, 24.3, and 12.24 U g^–1^ in autumn, respectively. These results demonstrated that the S-UE and S-SC activities were higher in spring than in autumn, but the S-CL activity was lower. Moreover, the S-UE and S-SC activities were higher and S-CL activity was lower at the V6 stage than the R6 in both seasons.

**TABLE 1:** Changes in soil enzyme activities with straw returning and nitrogen fertilization under dual-cropping system.

Season	Treatment	S-UE (U/g)		S-SC (U/g)		S-CL (U/g)	
		V6	R6	V6	R6	V6	R6
Spring	SR-N0	278.6 ± 12.2d	237.4 ± 12.1d	30.3 ± 0.2d	29.0 ± 0.6e	7.8 ± 0.0d	8.1 ± 0.0e
	SR-N100	310.6 ± 2.9c	246.2 ± 5.1cd	32.5 ± 0.1c	30.6 ± 0.2d	8.6 ± 0.1c	8.7 ± 0.0d
	SR-N150	326.4 ± 2.2bc	263.5 ± 2.4c	33.6 ± 0.2c	31.3 ± 0.4cd	8.8 ± 0.1c	9.3 ± 0.0c
	SR-N200	343.7 ± 7.5ab	293.3 ± 10.1b	35.7 ± 0.9b	32.4 ± 0.5bc	9.2 ± 0.1b	10.1 ± 0.1b
	SR-N250	353.6 ± 6.0a	304.7 ± 1.4ab	36.7 ± 0.8ab	33.8 ± 0.6ab	9.5 ± 0.1ab	10.4 ± 0.2ab
	SR-N300	354.3 ± 1.2a	318.7 ± 5.6a	38.5 ± 0.7a	34.5 ± 0.6a	9.7 ± 0.2a	10.7 ± 0.2a
	TP-N0	273.3 ± 13.4c	226.5 ± 5.1c	29.1 ± 0.7e	26.3 ± 0.7d	7.2 ± 0.2d	7.4 ± 0.0d
	TP-N100	301.5 ± 3.9c	241.0 ± 11.5bc	31.3 ± 0.8d	28.8 ± 0.8c	8.5 ± 0.1c	9.0 ± 0.1c
	TP-N150	317.7 ± 2.2b	246.7 ± 7.1bc	33.9 ± 0.9c	30.4 ± 0.2bc	8.7 ± 0.0bc	9.1 ± 0.0bc
	TP-N200	327.6 ± 4.9a	260.6 ± 3.9ab	34.4 ± 0.2bc	31.6 ± 0.9ab	8.9 ± 0.0ab	9.2 ± 0.0ab
	TP-N250	328.8 ± 2.3a	278.8 ± 4.5a	36.1 ± 0.4ab	33.3 ± 0.4a	9.0 ± 0.0a	9.3 ± 0.0a
	TP-N300	333.6 ± 4.8a	306.3 ± 11.8a	36.9 ± 0.5a	33.2 ± 0.7a	9.1 ± 0.1a	9.3 ± 0.0a
	P	NS	**	*	NS	NS	**
	N	**	**	**	**	**	**
	P × N	*	NS	NS	NS	**	**
Autumn	SR-N0	199.0 ± 1.5e	174.0 ± 0.9e	19.6 ± 0.2e	17.3 ± 0.5e	10.2 ± 0.1e	10.6 ± 0.3d
	SR-N100	215.9 ± 0.4d	185.3 ± 1.6d	22.5 ± 0.3d	20.9 ± 0.8d	11.4 ± 0.0d	11.5 ± 0.1c
	SR-N150	226.8 ± 1.5c	206.0 ± 1.3c	25.3 ± 0.6c	22.5 ± 0.4c	12.7 ± 0.0c	12.3 ± 0.1c
	SR-N200	243.7 ± 7.6b	232.4 ± 4.0b	30.3 ± 1.0b	29.5 ± 0.4b	13.7 ± 0.1b	14.3 ± 0.3bc
	SR-N250	249.2 ± 2.2ab	245.2 ± 4.2ab	32.2 ± 1.0ab	30.8 ± 0.1ab	14.1 ± 0.1ab	14.8 ± 0.2ab
	SR-N300	252.2 ± 1.4a	247.3 ± 0.2a	33.3 ± 0.5a	31.7 ± 0.3a	14.3 ± 0.3a	15.5 ± 0.5a
	TP-N0	193.4 ± 2.5d	162.9 ± 3.5d	15.2 ± 0.5d	10.6 ± 0.4c	7.9 ± 0.3d	9.6 ± 0.3d
	TP-N100	210.3 ± 2.9c	182.5 ± 0.4c	20.3 ± 0.8c	16.1 ± 0.5b	10.6 ± 0.1c	11.1 ± 0.1c
	TP-N150	219.5 ± 2.2b	195.8 ± 3.2b	23.2 ± 0.7b	17.3 ± 0.2b	11.6 ± 0.1b	11.9 ± 0.0b
	TP-N200	235.3 ± 3.7a	214.6 ± 0.8a	29.7 ± 0.7a	23.1 ± 0.4a	12.0 ± 0.2ab	12.7 ± 0.5a
	TP-N250	236.6 ± 0.5a	216.6 ± 0.5a	30.9 ± 0.6a	24.4 ± 0.4a	12.2 ± 0.2a	13.1 ± 0.0a
	TP-N300	242.0 ± 0.5a	219.5 ± 1.1a	31.4 ± 0.4a	24.6 ± 0.8a	12.3 ± 0.1a	13.4 ± 0.0a
	P	NS	*	NS	**	**	*
	N	**	**	**	**	**	**
	P × N	NS	**	*	NS	**	**

*Different letters indicate significant differences between samples (P < 0.05). Values are means ± SE (n = 3).*

*Significant at P < 0.05; **Significant at P < 0.01; NS, not significant.

*S–UE, soil urease activity; S–SC, soil sucrase activity; S–CL, soil cellulase activity. SR, TP, P, and N are straw return, traditional planting, planting pattern, and nitrogen, respectively.*

### Alpha Diversity of Soil Bacterial, Fungal, and Nematode Community

Based on the morphological traits, yield, soil organic carbon, nitrogen, and soil enzyme activities, highly significant differences were observed in response to 200 kg N ha^–1^ under both traditional and straw returning pattern. Therefore, the soil from the plot receiving 200 kg N ha^–1^ of maize field was adopted for soil bacterial, fungal, and nematode analysis.

After quality filtering, a total of 2,201 bacterial, 984 fungal, and 395 nematode OUTs were identified from 1415768, 1775268, and 1433535 RNA gene sequences, respectively. The bacterial OTUs number exhibited the trend SR-N0 > TP-N0 > SR-N200 > TP-N200 during spring and autumn seasons (Supplementary Figures 1A,B). Whereas the trend for soil fungal OTUs number was SR-N0 > SR-N200 > TP-N0 > TP-N200 during spring, and SR-N0 > TP-N0 > SR-N200 > TP-N200 during autumn (Supplementary Figures 2A,B), and the nematode OTUs number exhibited the trend TP-N200 > TP-N0 > SR-N200 > SR-N during spring, and during autumn the OTUs number for SR-N0 and TP-N200 was found to be the same; however, SR-N0 has higher OTUs than SR-N200 (Supplementary Figures 3A,B). The results indicated that the SR plot resulted in higher bacterial diversity (Shannon index) in the autumn season, however, it was not significantly affected by N fertilization. The bacterial richness (Chao and ACE index) was not affected by planting pattern or N fertilization, except SR-N0 had significantly higher ACE compared to TP-N200 in the autumn season (Table 2). The fungal richness (Chao and ACE index) was significantly affected by seasons, planting pattern, and N fertilization. During the spring season neither planting pattern nor N fertilization had significant effect on fungal diversity and richness, except SR-N0 treatment had significantly lower diversity compared to other treatments. Similarly, the fungal diversity was significantly higher in the SR treatment compared to TP during the autumn season, whereas SR-N0 had noticeably higher fungal richness than TP-N200 (Table 2). In contrast, the bacterial, fungal, and nematode richness was significantly higher in TP during the spring season compared to SR treatments. However, SR-N200 treatment had significantly higher nematode Chao1 and ACE richness compared to SR-N0 treatment during the spring season. Moreover, our results showed that the nematode diversity was not significantly affected by seasons, planting pattern, and N fertilization (Table 2). No variation in alpha-diversity of bacterial and nematode communities was observed during the spring season (*P* > 0.05). However, bacterial and fungal diversity were significantly higher in SR than TP treatments during the autumn season in response to N fertilization application suggesting that bacterial and fungal are more sensitive to straw returning than nematode in the autumn season (*P* < 0.05). The multi-factor analysis revealed that the bacterial (*P* < 0.05) and fungal alpha-diversity (*P* < 0.01) were significantly affected by planting pattern and seasons, respectively (Table 2).

**TABLE 2:** Alpha–diversity of the soil bacterial, fungal, and nematode communities with straw return and nitrogen application.

		Bacteria			Fungi			Nemotode		
		Diversity	Richness		Diversity	Richness		Diversity	Richness	
		Shannon	Chao	ACE	Shannon	Chao	ACE	Shannon	Chao	ACE
Spring	SR-N0	6.61 ± 0.02a	2101.6 ± 27.6a	2080.6 ± 32.8a	4.17 ± 0.08b	576.9 ± 75.9a	567.8 ± 69.3a	1.39 ± 0.36a	6.33 ± 2.31c	5.04 ± 5.06c
	SR-N200	6.65 ± 0.04a	2091.5 ± 28.6a	2073.4 ± 27.7a	4.30 ± 0.03a	565.8 ± 79.0a	556.7 ± 77.3a	1.48 ± 0.47a	14.67 ± 2.89b	15.04 ± 3.54b
	TP-N0	6.59 ± 0.06a	2096.4 ± 17.0a	2080.7 ± 23.6a	4.37 ± 0.06a	545.7 ± 78.8a	535.4 ± 77.1a	1.61 ± 0.62a	22.83 ± 8.31a	29.97 ± 15.71a
	TP-N200	6.54 ± 0.06a	2047.8 ± 45.0a	2038.7 ± 28.0a	4.30 ± 0.02a	460.6 ± 86.1a	458.1 ± 85.4a	1.92 ± 0.27a	21.50 ± 1.8a	22.46 ± 1.33a
Autumn	SR-N0	6.61 ± 0.04a	2116.1 ± 21.7a	2101.4 ± 17.6a	4.60 ± 0.09a	691.9 ± 9.6a	682.7 ± 13.6a	1.72 ± 0.21a	19.33 ± 4.04a	19.33 ± 4.04a
	SR-N200	6.64 ± 0.02a	2084.2 ± 26.8a	2067.3 ± 16.9ab	4.56 ± 0.07a	580.9 ± 80.6ab	575.5 ± 80.6ab	1.29 ± 0.44a	15.17 ± 6.45a	11.42 ± 13.47a
	TP-N0	6.62 ± 0.06ab	2111.6 ± 17.3a	2090.0 ± 21.0ab	4.41 ± 0.03b	588.7 ± 78.6ab	579.8 ± 82.0ab	1.42 ± 0.46a	20.33 ± 3.51a	20.76 ± 2.93a
	TP-N200	6.51 ± 0.03b	2069.9 ± 38.1a	2038.3 ± 26.0b	4.41 ± 0.05b	493.9 ± 108.3b	485.39 ± 105.4b	1.81 ± 0.16a	21.83 ± 2.02a	22.61 ± 1.93a
Planting pattern (P)		*	NS	NS	NS	*	*	NS	NS	NS
Nitrogen (N)		NS	*	*	NS	**	**	NS	NS	NS
Season (S)		NS	NS	NS	**	*	**	NS	NS	NS
P × N		*	NS	NS	NS	NS	NS	NS	NS	NS
P × S		NS	NS	NS	**	NS	NS	NS	NS	NS
N × S		NS	NS	NS	NS	NS	*	*	NS	NS
P × N × S		NS	NS	NS	NS	NS	NS	*	NS	NS

*Shannon, Chao, and ACE indexes were calculated based on phylogenetic distance at OTU level. Different letters indicate significant differences between samples (P < 0.05). Values are means ± SE (n = 3).*

*Significant at P < 0.05; ^**^Significant at P < 0.01; NS, not significant.

*SR, TP, P, N, and S are straw return, traditional planting, planting pattern, nitrogen, and season, respectively.*

### Beta Diversity of Soil Bacterial, Fungal, and Nematode Community

**FIGURE 2:**
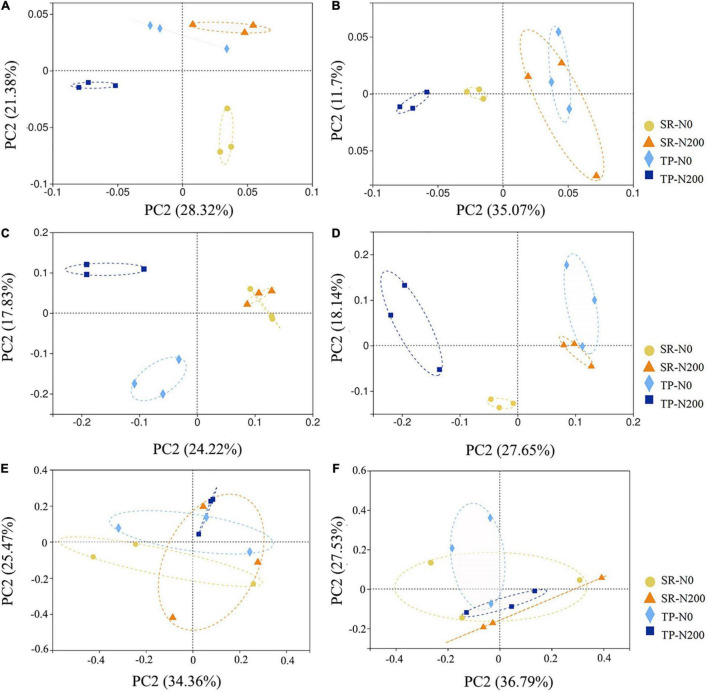
Beta diversities of soil bacterial during spring **(A)** and autumn **(B)**, fungal diversity in spring **(C)** and autumn **(D)**, and nematode diversity in spring **(E)** and autumn **(F)** seasons were analyzed by principal coordinates analysis (PCoA) based on unweighted Unifrac phylogenetic distance metrics at the OTU level and displayed in scatter diagram, each treatment with three replications. SR-N0 = straw returning without N fertilizer application; SR-N200 = straw returning with 200 kg ha^–1^ N fertilizer application; TP-N0 = traditional planting without N fertilizer application; TP-N200 = traditional planting with 200 kg/ha nitrogen fertilizer application.

The variations in bacterial, fungal, and nematode communities caused by SR and fertilization regime were explored using PCoA (Figure 2). The first two principal coordinates for the bacterial community represented 28.32 (PC1) and 21.38% (PC2) of total variation in spring (Figure 2A), and 35.07 (PC1) and 11.70% (PC2) in autumn maize fields (Figure 2B). These results demonstrated that the beta-diversity of bacterial community structure was significantly affected by planting pattern and N fertilization during the spring season; however, during the autumn season the SR-N200 and TP-N0 were overlapped and showed non-significant effect on soil bacterial community.

To assess the fungal beta diversity, the phylogenetic analysis of fungal composition was performed using unweighted UniFrac distances in spring (Figure 2C) and autumn (Figure 2D). The results of the current study revealed that autumn has more comparable community structure than the spring season. The PC1 and PC2 principal coordinates explained 24.2 and 17.8% of variation in spring and 27.7 and 18.1% of variation in autumn, respectively (Figures 2C,D). The application of N fertilizer in the spring altered the structure of the fungal community in a positive direction of PC2. The correlation between SR-N200 and SR-N0 treatments during both seasons was closer, while significant separation was observed for TP-N200 and TP-N0 treatments (Figures 2C,D). These results revealed that the fungal beta-diversity were strongly affected by both planting pattern and N fertilization during the autumn season (Figure 2D); however, the SR-N200 and SR-N0 treatments were not significantly different during the spring season (*P* > 0.05; Figure 2C).

The nematode community analysis indicated that the first and second principal coordinates represented 34.36 and 25.74% of the variation in spring (Figure 2E), and 36.79 and 27.53% of the variation in autumn, respectively (Figure 2F). The PCoA results indicated that during the spring season the SR-N200 treatment was not significantly different from other treatments. However, SR-N0 was significantly different from TP-N0 and TP-N200 treatments (*P* < 0.05; Figure 2E). During the autumn season the treatment SR-N0 was non-significant from the other treatments, but SR-N200 treatment was significantly separated from TP-N0 and TP-N200 treatments (*P* < 0.05; Figure 2F). These results suggest that the nematode community was not significantly affected by N fertilization, however, planting patterns have significant effect in both spring and autumn seasons.

### Relative Abundance and Community Compositions

#### Soil Bacterial Abundance and Community Composition

**FIGURE 3:**
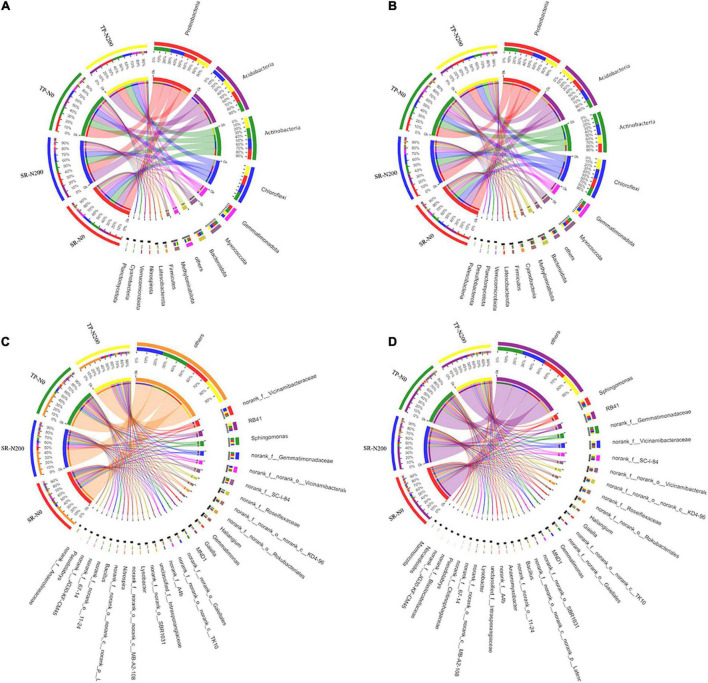
Relative abundances of bacterial in spring **(A)** and autumn **(B)** communities at the phylum level, and abundance at the genes level in spring **(C)** and autumn **(D)** seasons. SR-N0 = straw returning without N fertilizer application; SR-N200 = straw returning with 200 kg ha^–1^ N fertilizer application; TP-N0 = traditional planting without N fertilizer application; TP-N200 = traditional planting with 200 kg/ha nitrogen fertilizer application.

In all treatments, including seasons, planting pattern, and nitrogen fertilizer application, 30 bacterial phyla were identified. In spring, 14 of the most abundant phyla were identified, accounting for 96.51–97.15% of all sequences (Supplementary Figure 4A). The microbial population was dominated by Proteobacteria (20.90–25.89%), followed by Acidobacterioia (18.56–22.47%), Chloroflexi (13.32–15.42%), and Actinobacteriota (16.41–16.79%). In autumn, 15 of the most abundant phyla were identified, accounting for 96.73–97.64% of all sequences (Supplementary Figure 4B). Like spring, the dominant bacterial phyla were Proteobacteria (21.06–23.26%), Acidobacterioia (13.68–23.88%), Chloroflexi (13.72–16.24%), and Actinobacteria (13.83–17.74%) in the autumn. In the SR-N0, SR-N200, TP-N0, and TP-N200 soils, Proteobacteria, Acidobacteriota, Actinobacteriota, and Chloroflexi were the top four most abundant bacterial phyla irrespective of planting seasons (Figures 3A,B). In SR-N200 soil, the Proteobacteria (22%), Acidobacteriota (22%), Actinobacteriota (16%), and Chloroflexi (14%) were the top four most abundant phyla in the spring planting pattern (Figure 3A). The abundance of Actinobacteriota and Chloroflexi were noticeably increased, whereas the abundance of Proteobacteria was markedly decreased in the TP-N200 soil compared to SR-N200 soil. In addition, the Proteobacteria and Actinobacteriota were potentially decreased, whereas the abundance of Acidobacteriota and Chloroflexi were increased in the SR-N0 soil compared to TP-N0 (Figure 3A). These results suggest that soil with SR and optimum N fertilization (200 N kg ha^–1^) presents a significantly greater abundance of Proteobacteria, Acidobacteriota, and Chloroflexi in the spring season. Similarly, in the autumn season, the Proteobacteria, Acidobacteriota, Actinobacteriota, and Chloroflexi were the top four most abundant phyla in SR-N200 and TP-N200 soils (Figure 3B). The results of our current study suggest that the Acidobacteriota (24%) and Chloroflexi (16%) were markedly increased in the TP-N200 soil, but the abundance of Actinobacteriota (14%) was decreased compared to SR-N200 soil (Figure 3B).

At the genus level, the bacterial community structures showed significant differences among the different treatment and seasons (Figures 3C,D). For example, during the spring season in TP-N200 soil, the most dominant genera were norank_f_*Vicinamibacteraceae* (4.7%), RB41 (5.9%), and *Sphingomonas* (3.9%). Furthermore, suggesting that the TP-N0 soil significantly decreased the norank_f_*Vicinamibacteraceae* (3.3%) and *RB41* (4.1%), however, it increased the abundance of *Sphingomonas* (5%). Likewise, in the SR-N200 and SR-N0 soils the dominant bacterial genus was norank_f_*Vicinamibacteraceae* (6.9 and 4.1%), RB41 (3.4 and 3.8%) and *Sphingomonas* (3.2 and 4.1%), respectively, suggesting that the *RB41* and *Sphingomonas* were significantly dominant in SR-N0 soil compared to SR-N200 soil. During the autumn season, the TP-N200 soil has the highest abundance of genera *Sphingomonas* (4.8%) and *RB41* (5.8%) compared to other treatments (Figure 3D). In addition, the abundance of *Sphingomonas* and *RB41* were significantly increased in TP having 200 N ha^–1^ than soil with no fertilization, however, the SR-N200 soil decreased the abundance of these genera compared to SR-N0 soil. These results indicated that the abundances of genera *Sphingomonas* and *RB41* were significantly decreased with N fertilization in an SR plot, however, they decreased under a TP plot (Figure 3D).

#### Soil Fungal Abundance and Community Composition

**FIGURE 4:**
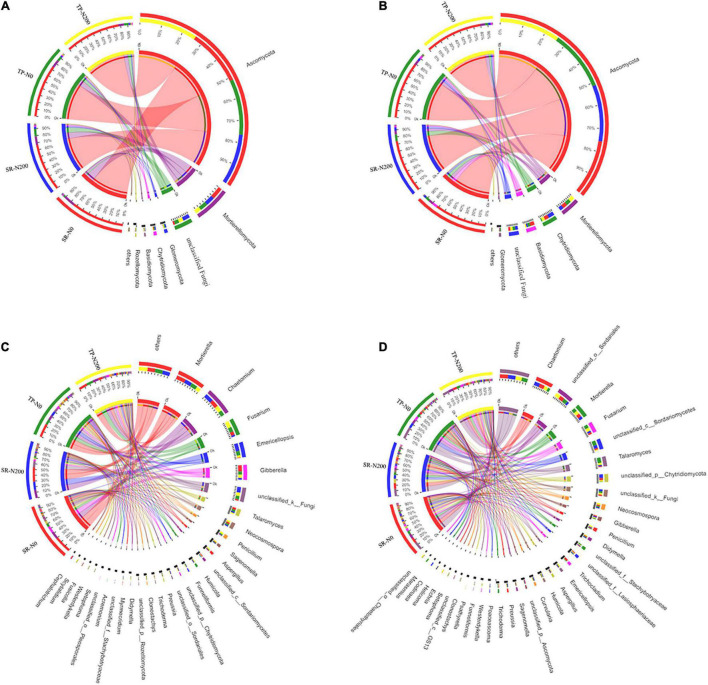
Relative abundances of fungal in spring **(A)** and autumn **(B)** communities at the phylum level, and abundance at the genes level in spring **(C)** and autumn **(D)** seasons. SR-N0 = straw returning without N fertilizer application; SR-N200 = straw returning with 200 kg ha^–1^ N fertilizer application; TP-N0 = traditional planting without N fertilizer application; TP-N200 = traditional planting with 200 kg/ha nitrogen fertilizer application.

Various fungal phyla, including Ascomycota, Mortierellomycota, Chytridiomycota, Glomeromycota, Basidiomycota, and unclassified-fungal phyla, were found in all treatments, regardless of season, planting pattern, or nitrogen fertilizer application; however, Rozellomycota was only found in the spring season (Supplementary Figures 5A,B). Ascomycota, Mortierellomycota, and Chytridiomycota were found to be the most abundant fungal phyla in both seasons (Figures 4A,B and Supplementary Figures 5A,B). The phyla Ascomycota (75%) have the highest relative abundance among the fungal community, followed by Mortierellomycota (12%), unclassified (5.39%), and Chytridiomycota (1.75%). Our findings demonstrated that SR treatments have variable degrees of impact on the phylum-level composition of fungal communities. In the TP-N200 and SR-N200 soils, the Ascomycota (79 and 69%), Mortierellomycota (8.2 and 13%), and unclassified-fungi (6.7 and 11%) were the top three most abundant phyla in the spring season, respectively (Figure 4A). These results suggest that the abundance of Ascomycota decreased in SR-N200 soil, while Mortierellomycota and unclassified-fungi significantly increased compared to TP-N200 soil. In addition, the relative abundance of Ascomycota and Mortierellomycota were potentially increased, whereas the abundance of unclassified-fungi and Glomeromycota were decreased in the SR-N0 soil compared to TP-N0 (Figure 4A). These results demonstrated that SR increased the relative abundance of Ascomycota, Mortierellomycota, and Glomeromycota regardless of N fertilization. Similarly, in SR-N200 soil during autumn season, Ascomycota, Mortierellomycota, Chytridiomycota, and Basidiomycota were 76, 5.5, 9.4, and 2.5%, respectively (Figure 4B). However, in TP-N200 soil the relative abundance of Ascomycota, Mortierellomycota, Chytridiomycota, and Basidiomycota were 81, 7.3, 4.4, and 2.1%, respectively. The above results showed that the abundance of Ascomycota and Mortierellomycota was noticeably increased in TP-N220 soil whereas it was decreased in Chytridiomycota and Basidiomycota (Figure 4B).

At the genus level, the fungal community structure showed that *Mortierella*, *Chaetomium*, *Fusarium*, *Emericellopsis*, and *Gibberrella* were the most abundant genera in all the treatments (Figure 4C). The abundance of *Mortierella, Emericellopsis*, and *Gibberrella* was noticeably decreased in TP-N0 soil compared to TP-N200 soil, however, *Chaetomium* and *Fusarium* were increased. Likewise, in SR-N200 and SR-N0 soils the relative abundance of the top five genera were *Mortierella* (13 and 17%), *Chaetomium* (15 and 14%), *Fusarium* (3 and 6.4%), *Emericellopsis* (15 and 1%), and *Gibberrella* (3.5 and 12%), respectively. In the autumn season, the highest relative abundance of Ascomycota was observed in TP-N200 soil (81%), TP-N0 soil (81%), SR-N200 soil (76%), and SR-N0 soil (75%). Furthermore, the abundance of genera *Ascomycota* and *Mortierellomycota* were increased in the TP pattern, whereas the genre *Chytridiomycot* and *Basidiomycota* were decreased compared to SR (Figure 4D).

#### Soil Nematode Abundance and Community Composition

**FIGURE 5:**
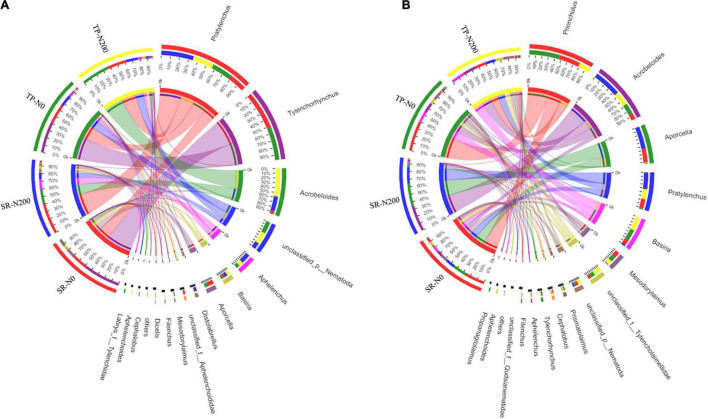
Relative abundances of nematode at the genes level in spring **(A)** and autumn **(B)** seasons. SR-N0 = straw returning without N fertilizer application; SR-N200 = straw returning with 200 kg ha^–1^ N fertilizer application; TP-N0 = traditional planting without N fertilizer application; TP-N200 = traditional planting with 200 kg/ha nitrogen fertilizer application.

It was found that there were 15 different types of nematode genera in total during the spring season, and the most abundant genera were *Pratylenchus*, *Tylenchorhynchus*, *Acrobeloides*, *unclassified*, *Aphelenchus*, *Basiria*, and *Aporcella* (Supplementary Figure 6A). The dominant nematode genera in TP-N200, TP-N0, SR-N200, and SR-N0 were *Acrobeloides* (37%), *Tylenchorhynchus* (42%), *Pratylenchus* (42%), and *Tylenchorhynchus* (50%), respectively. In addition, the abundance of *Pratylenchus* (27%) and *Acrobeloides* (37%) were increased in TP-N200 soil but decreased the *Tylenchorhynchus* (0.028%) compared to TP-N0 soil and vice versa (Figure 5A). Similarly, the nematode genera *Pratylenchus, Acrobeloides*, and *Tylenchorhynchus* accounted for 42, 19, and 0% of the total abundance in SR-N200 soil, respectively. However, the relative abundance of *Tylenchorhynchus* was significantly higher in both TP-N0 and SR-N0 soils compared to the TP-N200 and SR-N200, suggesting that N fertilization negatively affected the abundance of *Tylenchorhynchus* (Figure 5A). In the autumn season, the most abundant nematode genera were *Prionchulus*, *Acrobeloides, Aporcella, Pratylenchus, Basiria*, and *Mesodorylaimus* (Figure 5B and Supplementary Figure 6B). The dominate genera in TP-N200, TP-N0, SR-N200, and SR-N0 were *Basiria* (19%), *Prionchulus* (40%), *Acrobeloides* (36%), and *Prionchulus* (28%). These results showed that the *Prionchulus* was significantly increased in an unfertilized plot; however, the *Aprocella* compared to an N fertilized plot decreased. Moreover, the relative abundance of genera *Tylenchorhynchus* and *Aphelenchus* was found significantly higher in the spring seasons, but significantly less abundance was observed in the autumn seasons (Figures 5A,B).

### Relationships Between Bacterial, Fungal, and Nematode Community and Soil Environmental Properties

**FIGURE 6:**
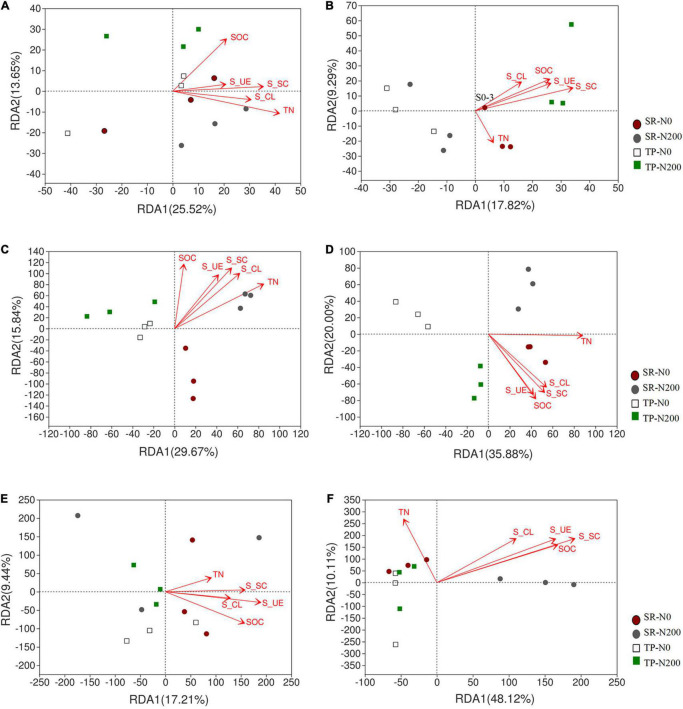
The redundancy analysis (RDA) to identify the relationship of bacterial fungal and nematode OTUs taxa and soil properties. Bacterial in spring **(A)** and autumn **(B)**, fungal in spring **(C)** and autumn **(D)**, and nematode in spring **(E)** and autumn **(F)** seasons. S0 = straw returning without nitrogen fertilizer application; S200 = straw returning with 200 kg/ha nitrogen fertilizer application; T0 = traditional planting without nitrogen fertilizer application; T200 = traditional planting with 200 kg/ha nitrogen fertilizer application.

**FIGURE 7:**
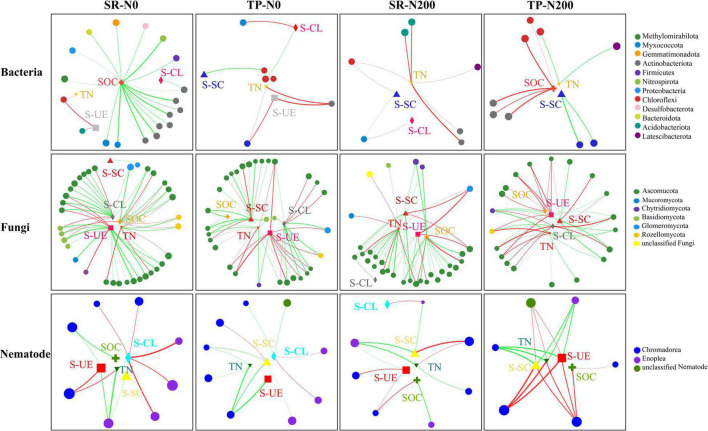
Co-occurrence network diagram between bacterial and fungal taxa at the phyla level and nematode on the class level with soil fertility and enzymes under different treatments. Dot size indicates the value of relative abundances. Positive correlations are labeled with red lines and negative correlations are colored in green, thick lines are high correlations and little correlations have thin lines. Treatments: no nitrogen addition + straw addition (SR-N0), no nitrogen addition + traditional planting (TP-N0), no nitrogen addition + straw addition (SR-200), and traditional planting with nitrogen fertilizer application (TP-N200). TN, SOC, S-UE, S-CL, and S-SC represent total nitrogen content, soil organic carbon content, soil urease activity, soil sucrase activity, and soil cellulase activity.

A multivariate RDA analysis indicated that soil environmental variables such as SOC, TN, S-UE, S-CL, and S-SC contributed to the distribution of the bacterial, fungal, and nematode OTUs (Figure 6). The first two RDA axes explained 39.17 and 27.11% variation in spring (Figure 6A) and autumn (Figure 6B), respectively. In addition, the relationship among the bacterial species and environmental factors, suggesting 25.52 and 13.65% variation in spring, and 17.82 and 9.29% variation in autumn for the first and second axes, respectively. The correlation analysis showed that the S-CS, TN, and S-UE significantly increased the bacterial phyla FCPU426 (*P* < 0.01) and significantly decreased Cyanobacteria (*P* < 0.05; Supplementary Figure 7). Moreover, S-CL significantly decreased FCPU426 (*P* < 0.001) and Nitrospirota (*P* < 0.05), whereas SOC, S-CS, and S-UE increased Acidobacteriota. The relationship between changes in environmental factors and the relative abundances of fungal OTUs is shown in Figures 6C,D. In spring and autumn, the SOC, TN, S-UE, S-CL, and S-SC accounted for 45.51 and 55.88% of the overall variation, respectively. Furthermore, these results clearly demonstrated that the first and second axes account for 29.67 and 15.84% variation in spring, and 35.88 and 20.00% variation in autumn, respectively. Similarly, soil enzymes significantly affected the fungal phyla, the correlation analysis revealed that S-CL increased the abundance of Chytridiomycota and Basidiomycota (*P* < 0.001), while decreased Mucoromycota and Rozellomycota. In contrast, the abundance of Mucoromycota and Rozellomycota increased with TN and S-UC (Supplementary Figure 8). The first two axes suggested that environmental variables such as SOC, TN, S-UE, S-CL, and S-SC strongly influenced the distribution of the nematode OTUs. Redundancy analysis was used to understand the relationship between the nematode community and soil environmental characteristics (Figures 6E,F). These results demonstrated that the first two axes of RDA accounted for 26.04 and 10.89% variation in the spring season (Figure 6E), while 33.24 and 11.15% in the autumn season (Figure 6F). Soil enzyme activities, SOC, and TN were strongly associated with the nematode community distribution in TP-N200 and SR-N200 treatments during the spring season. However, soil enzymes and SOC were associated with SR-N0 and SR-N200 treatment in the autumn season, but TN was nearly correlated to TP-N0. The nematode genera *Pratylenchus* and unclassified *Aphelenchoididae* have positive correlation, while unclassified *Tylencholaimellidae*, *Prismatolaimus*, *Prionchulus*, and *Mesodorylaimus* have negative correlation with S-CS, TN, and S-UE (Supplementary Figure 9). The co-occurrence network diagram further clarified the specific changes in soil fertility and correlation with bacterial, fungal, and nematode community structure under different planting patterns and N fertilizers (Figure 7). These results showed that S-SC has a positive relationship with bacterial phyla Chlorflexi, S-CL with Proteobacteria, and TN with Chloroflexi, Acidobacteria, and Actinobacteriota in SR-N200 soil. However, SOC has a positive relation with Chloroflexi and Actinobacteriota in TP-N200. The fungi and nematode have much more strong and positive correlation with soil enzymes, SOC, and TN in TP-N200 compared to SR-N200.

## Discussion

### Changes in Soil Fertility and Enzyme Activities With Planting Pattern and N Fertilization

Soil organic carbon and N are the primary sources of energy and nutrients for microbial growth (Zhao et al., 2016). The SOC is derived from incorporated crop residues and rhizodeposited organic matter (Banger et al., 2010; Yang et al., 2012). Incorporated residues are decomposed rapidly and returned large amounts of N and C into the field, which lead to improve soil quality and boosted soil fertility (Liu et al., 2014). In addition, the application of N fertilizers increases soil TN and SOC compared to the control treatment (N0). Averaged across fertilization, the SR significantly increases SOC and TN during both seasons compared to the TP pattern (Figure 1). However, the spring season has a much higher SOC and TN content than that of the autumn season under both planting patterns. These findings showed that improved SOC and TN contents are mostly attributable to spring maize having more time to decompose the maize straw; however, straw obtained after spring maize harvesting had less time to decompose for the autumn maize (Liao et al., 2018; Zhao et al., 2021). These findings are in line with findings from earlier research (Chao et al., 2019), they also demonstrated that spring maize has higher soil fertility compared to autumn maize, which might be due to the straw incorporated into the soil having the proper time to decompose before sowing.

The soil enzymatic activities boosted in response to chemical fertilizer application and SR due to greater C demand for microbial development under high N content (Liu et al., 2021). Soil enzyme activity is more important in evaluating soil quality and plays an important role in nutrient recycling (Demisie et al., 2014). The increase in soil enzyme activities (Table 1) could be explained by changes in the microbial community composition, as well as improved soil microorganism metabolism and microbial activity stimulation by straw addition (Iovieno et al., 2009). Therefore, SR can produce organic supplements and increase microbial biomass, delivering sufficient energy and establishing an appropriate environment for the development of soil enzymes (Gianfreda et al., 2005).

### Straw Returning Increases Bacterial, Fungal, and Nematode Diversity

Soil microbes, such as bacteria and fungi, have a major part in the decomposition of straw. Bacteria have a faster growth rate than fungi and play a bigger part in the early phases of decomposition while the fungi, on the other hand, play a bigger role in later stages of decomposition (Marschner et al., 2011; Wang L. et al., 2021). Straw returning increases the bacterial and fungal diversity during the autumn season; thereby changing the structure of the soil ecosystem and making it more stable and healthier (Yin et al., 2010). In the current study we find that the SR plot significantly increases the Shannon index of bacterial and fungal communities during the autumn season. Furthermore, Chao and ACE index of bacterial and fungal communities are significantly higher in SR-N0 than that of the TP-N200 treatment (*P* < 0.05; Table 2). These results suggest that SR improves soil bacterial and fungal diversity and richness. This may be attributed to the rapid growth rate of bacteria, which promotes straw decomposition in the early stages associated with a larger number of nematodes diversity in the SR plot (Zhang et al., 2012; Wang G. et al., 2021).

Our study finds that SR treatments significantly reduce soil nematode richness and diversity compared to TP treatments in the spring season, but SR-N200 has significantly higher soil nematode Chao and ACE indexes than that of the SR-N0 treatment (Table 2). Our findings are consistent with the findings of earlier researchers (Ferreira et al., 2021), who reported that fungal diversity and richness were unaffected by fertilization. Chemical fertilization, on the other hand, reduced bacterial diversity and richness, according to Feng et al. (2015). These results were also confirmed by Zhou et al. (2015), who found that long-term use of a single fertilizer reduces the diversity and richness of the soil bacterial community. In north China, the long-term uses of chemical and organic fertilizers increase the population of bacteria and fungi as reported by Li et al. (2017). As a result, straw addition with N fertilizer application have a key role in sustaining the diversity of soil bacteria, fungi, and nematode communities, which in turn may help to prevent the degradation of the soil microbial community structure and function over time (Sun et al., 2015).

### Effect of Straw Returning on Bacterial, Fungal, and Nematode Communities Decomposition

The soil microbial community structure could be altered by SR and N fertilization (Li et al., 2017; Zhao et al., 2019). In this study, Proteobacteria, Acidobacteria, Nitrospirae, Chloroflexi, and Actinobacteria are the important components of the soil bacterial community (Supplementary Figures 3A,B), these results are supported by a previous study (Liu et al., 2020). However, the current study found no significant difference in the relative abundance of major soil bacteria phyla between SR and TP plots. The high number of Ascomycota taxa could be attributed to their easy colonization and rapid growth (Liu et al., 2017; Wang L. et al., 2021). In addition, SR has a considerable impact on the abundance of several fungi. Average abundances of fungal phyla Mucoromycota, Chytridiomycota, unclassified fungi, and Rozellomycota differ greatly in SR plots as compared to TP. Even though there were many different phyla in the soil, some of them were not dominating and their abundance was extremely low. In the SR plot, the relative abundances of Rozelloomycoa varied, these results are in line with recent findings (Fan and Wu, 2020; Wang G. et al., 2021). *Pratylenchus*, *Tylenchorhynchus*, *Prionchulus*, and *Acrobeloides* were dominant and important genera of the soil nematode community (Figures 5A,B).

The bacterial and fungal community compositions are changed with N fertilization, planting pattern, and seasons. The most dominant bacterial phyla are Proteobacteria and Acidobacteria, and dominant fungal phyla were Ascomycota, Mucoromycota, and Chytridiomycota in both planting pattern and seasons. Furthermore, the phyla Chytridiomycota, unclassified fungi, and Glomeromycota are significantly increased in N200 kg N ha^–1^ compared to N0 irrespective of the planting pattern. Similarly N fertilizer application has significantly positive effects on the nematode abundances (Supplementary Figure 3). The abundance of genera *Pratylenchus* and *Acrobeloides* is noticeably increased in fertilized plots (N200 kg N ha^–1^) compared to N0 treatment during both seasons. According to the finding of Allison and Martiny (2008), who reported that out of 38 publications 84% of studies suggest that the soil microbial communities are more sensitive to fertilization especially N.

### Relationships of Bacterial, Fungal, and Nematode Community With Soil Fertility and Enzyme Activities

The Actinobacteria was the most dominant phylum of bacteria in SR-N0 compared to TP-N0, indicating that it is more prominent in straw decomposition than other microbial fractions (Chen et al., 2016; Li et al., 2017). Moreover, SR-N200 increases bacterial Acidobacteria at the phyla level compared to TP-N200. Different bacterial community compositions suggested that bacteria dominates soil C decomposition, these changes in bacterial fractions suggested that bacteria are important in the degradation of straw and the activity of enzymes (Zhao et al., 2016). The microbial community composition varies in plant growth, but also in reaction to the soil environment in the field, including nitrogen, organic carbon, and enzyme activity (Mouhamadou et al., 2013; Carrara et al., 2018; Hou et al., 2020). Our results show that the changes in the bacterial composition of the soil are identified in response to planting pattern and are closely related to N fertilizer application (Figure 6). Furthermore, the increase in diversity of bacterial composition in response to N fertilization is mostly attributable to changes in the relative abundance of certain bacterial phyla such as Chloroflexi, Actinobacteria, Acidobacteria, and Latescibacterota. The co-occurrence network analysis found that the abundance of Chloroflexi and Acidobacteria was closely related to TN in different planting patterns (Figure 7). These results are in line with the findings of other researchers (Fierer et al., 2007), who reported that these bacterial phyla have been linked to the soil C and N cycles.

Changes in the soil fungal community in response to SR could be more directly linked to changes in SOC and N derived from straws. As several different types of fungi are saprotrophic in the soil, acquiring SOC, N, and energy from incorporated crop residues (Su et al., 2020b). This might be one reason for the results of RDA that soil enzymes, SOC, and TN were found to be significantly correlated with soil fungal community variation (Figures 6C,D). These results further suggest that the most of soil enzymes and soil nutrients are significantly correlated with SR-N200 during spring and with SR-N0 during autumn. The current study results show that the fungal phylum Ascomycota is the most abundant phylum in SR-N0 compared to TP-N0, suggesting that Ascomycota predominated in the SR plot compared to other microbial segments (Ma et al., 2013; Yang et al., 2016). Additionally, at the phylum level, SR-N200 increased the fungal Chytridiomyeota division. Soil enzymes are essential for decomposing soil organic matter and unique to catalyze a specific biochemical reaction (Li et al., 2016). RDA results show differences in the enzymes affecting the changes of the nematode community, which is mainly influenced by S-UE and S-SC in spring (Figure 6E) and autumn (Figure 6F), respectively. Moreover, S-UE and S-CL had a positive impact on nematode communities at the genus level in SR-N0 and SR-N200 plots, and S-SC, S-UE, and SOC had positive impact in a TP-N200 plot (Figure 5).

## Conclusion

This study investigates the impact of straw returning and N fertilizer on the composition and diversity of bacterial, fungal, and nematode communities, as well as their effects on soil enzymes, SOC, and TN of soil under maize plantation. The SOC, TN, S-UE, S-SC, and S-CL activities in the SR plot following N fertilization increase specifically at the V6 stage than the R6 in an SR plot. The SR incorporation is an important determinant in influencing the composition of the bacterial and fungal communities and improved soil fertility compared to TP. The most dominant soil bacterial and fungal species in spring and autumn were Proteobacteria and Ascomycota, and the most dominant nematode genera were *Pratylenchus* and *Prionchulus* in spring and autumn, respectively. RDA and co-occurrence network identified that TN, SOC, and S-SC were closely correlated with bacterial and fungal community composition. These findings will be helpful in framing the straw returning and N fertilizer application for improving soil fertility, bacterial, and fungal structure and communities on sustainable way and minimizing the degradation of soil in sub-tropical regions of China.

## Data Availability Statement

The datasets presented in this study can be found in online repositories. The names of the repository/repositories and accession number(s) can be found at: NCBI – PRJNA803963.

## Author Contributions

LY and XZ designed the experiment. LY performed the experiment and wrote the manuscript. YC and IM helped in data collection. DW data curation. IM writing–review and editing. XZ resources and supervision. All the authors have read and approved the final manuscript, agreed to the published version of the manuscript.

## Conflict of Interest

The authors declare that the research was conducted in the absence of any commercial or financial relationships that could be construed as a potential conflict of interest.

## Publisher’s Note

All claims expressed in this article are solely those of the authors and do not necessarily represent those of their affiliated organizations, or those of the publisher, the editors and the reviewers. Any product that may be evaluated in this article, or claim that may be made by its manufacturer, is not guaranteed or endorsed by the publisher.

## Footnotes

1
https://cran.r-project.org/web/packages/rda/
