# Supplementary material for: Straw Return and Nitrogen Fertilization to Maize Regulate Soil Properties, Microbial Community, and Enzyme Activities Under a Dual Cropping System
Source: Front Microbiol. 2022 Mar 15;13:823963. doi: 10.3389/fmicb.2022.823963 (PMC8965350; doi:10.3389/fmicb.2022.823963)
Supplement: Supplementary file 1 [file Data_Sheet_1.docx]

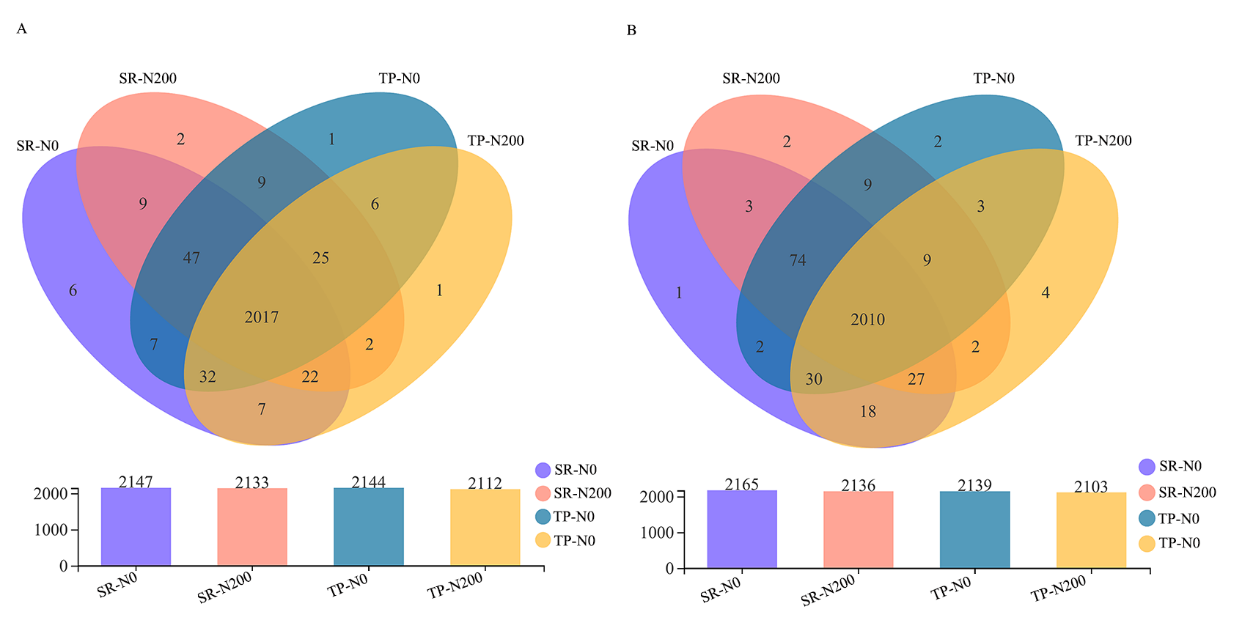


**Figure S1.** Venn of soil bacterial during spring (A) and in autumn (B) seasons.

Abbreviations: SR-N0 = straw returning without N fertilizer application; SR-N200 = straw returning with 200 kg ha^-1^ N fertilizer application; TP-N0 = traditional planting without N fertilizer application; TP-N200 = traditional planting with 200 kg/ha nitrogen fertilizer application.


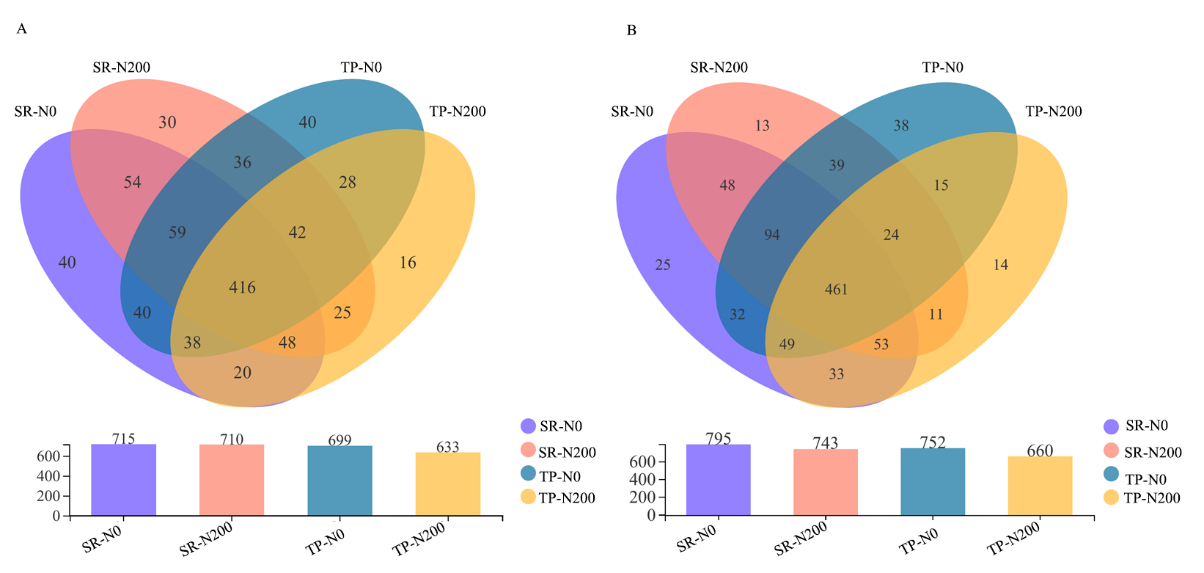


**Figure S2.** Venn of soil fungi during spring (A) and in autumn (B) seasons.

Abbreviations: SR-N0 = straw returning without N fertilizer application; SR-N200 = straw returning with 200 kg ha^-1^ N fertilizer application; TP-N0 = traditional planting without N fertilizer application; TP-N200 = traditional planting with 200 kg/ha nitrogen fertilizer application.


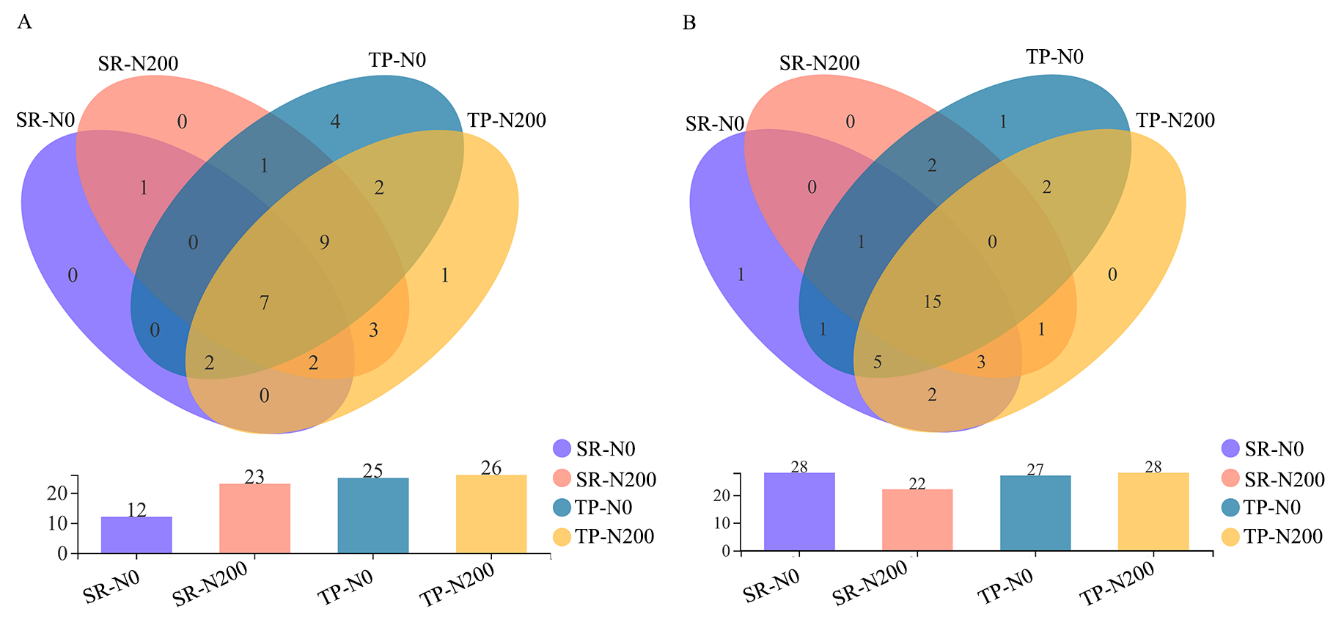


**Figure S3.** Venn of soil nematode during spring (A) and in autumn (B) seasons.

Abbreviations: SR-N0 = straw returning without N fertilizer application; SR-N200 = straw returning with 200 kg ha^-1^ N fertilizer application; TP-N0 = traditional planting without N fertilizer application; TP-N200 = traditional planting with 200 kg/ha nitrogen fertilizer application.


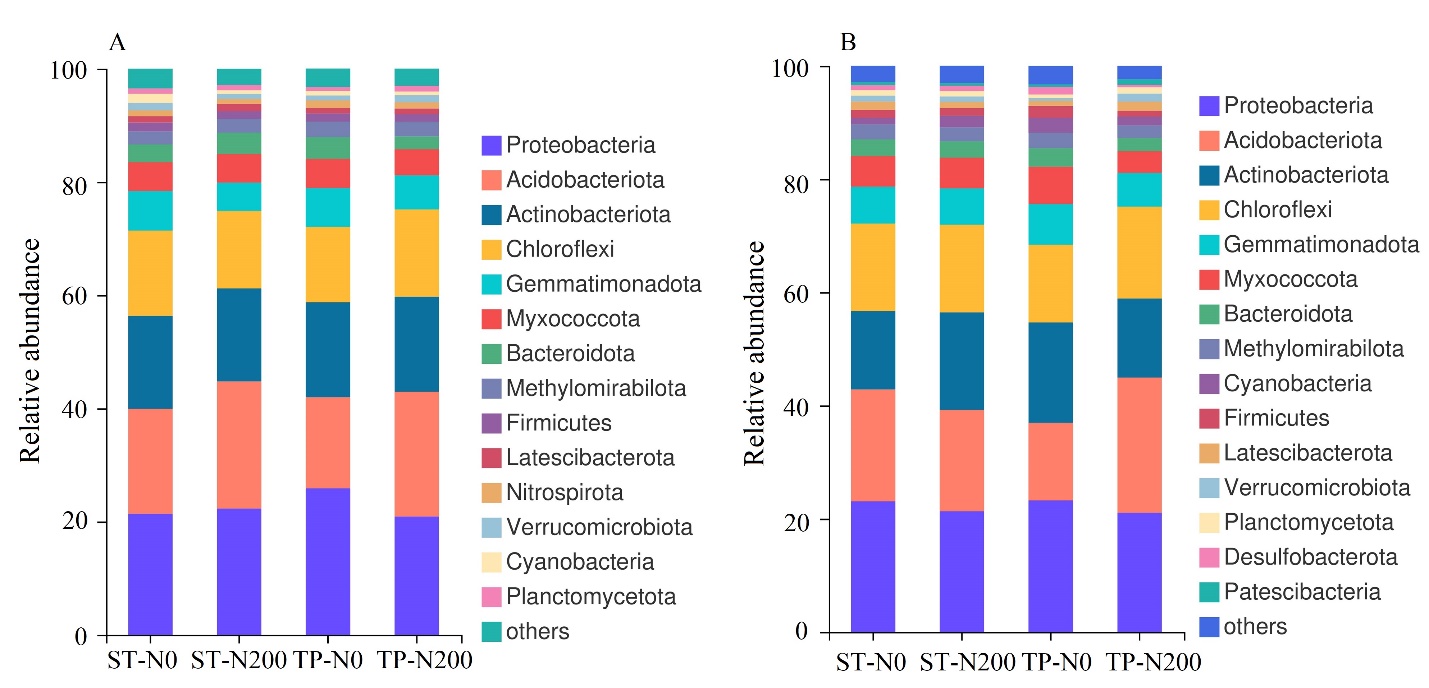


**Figure S4.** Relative abundance of soil bacterial during spring (A) and in autumn (B) seasons.

Abbreviations: SR-N0 = straw returning without N fertilizer application; SR-N200 = straw returning with 200 kg ha^-1^ N fertilizer application; TP-N0 = traditional planting without N fertilizer application; TP-N200 = traditional planting with 200 kg/ha nitrogen fertilizer application.


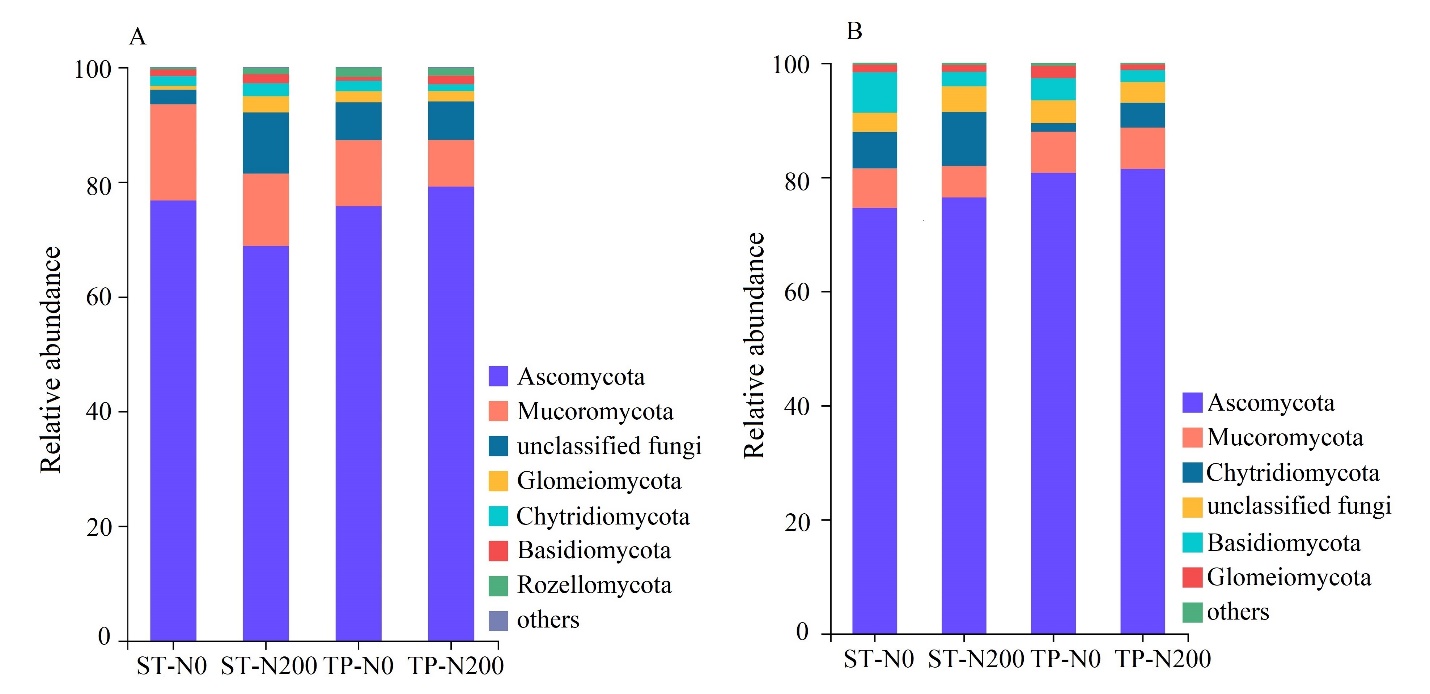


**Figure S5.** Relative abundance of soil fungi during spring (A) and in autumn (B) seasons.

Abbreviations: SR-N0 = straw returning without N fertilizer application; SR-N200 = straw returning with 200 kg ha^-1^ N fertilizer application; TP-N0 = traditional planting without N fertilizer application; TP-N200 = traditional planting with 200 kg/ha nitrogen fertilizer application.


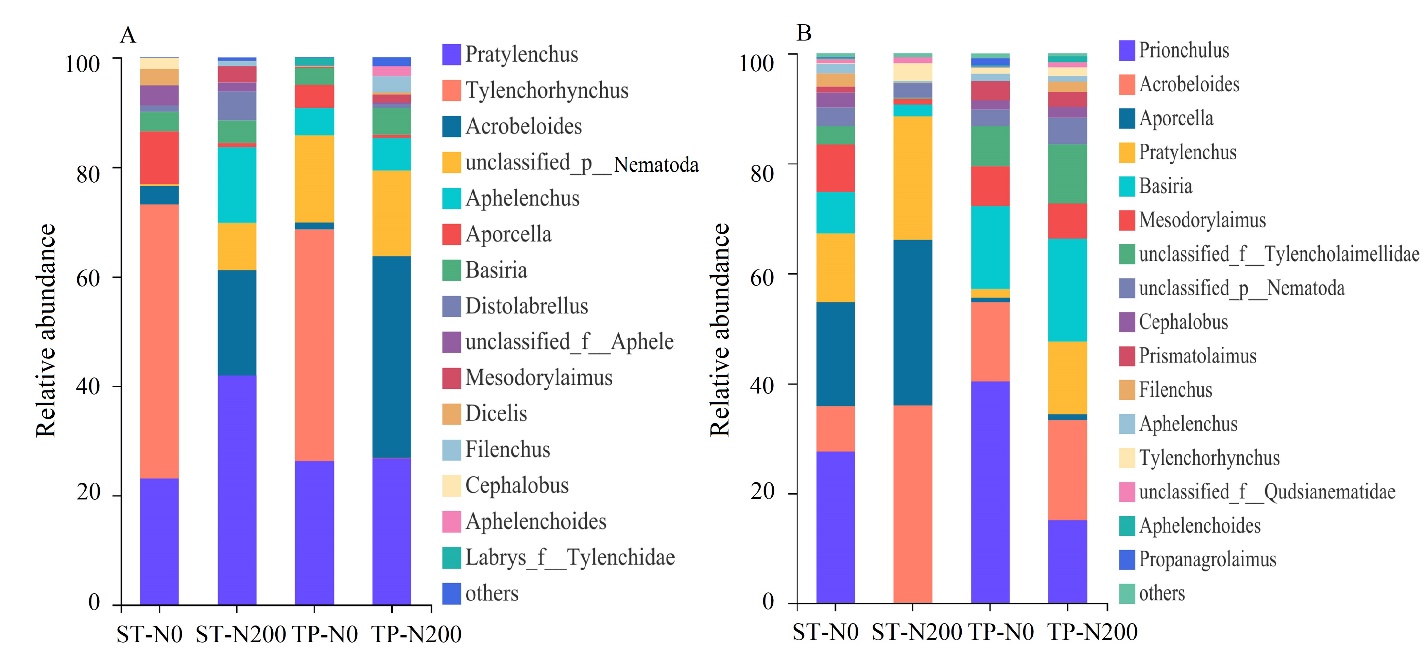


**Figure S6.** Relative abundance of soil nematode during spring (A) and in autumn (B) seasons.

Abbreviations: SR-N0 = straw returning without N fertilizer application; SR-N200 = straw returning with 200 kg ha^-1^ N fertilizer application; TP-N0 = traditional planting without N fertilizer application; TP-N200 = traditional planting with 200 kg/ha nitrogen fertilizer application.


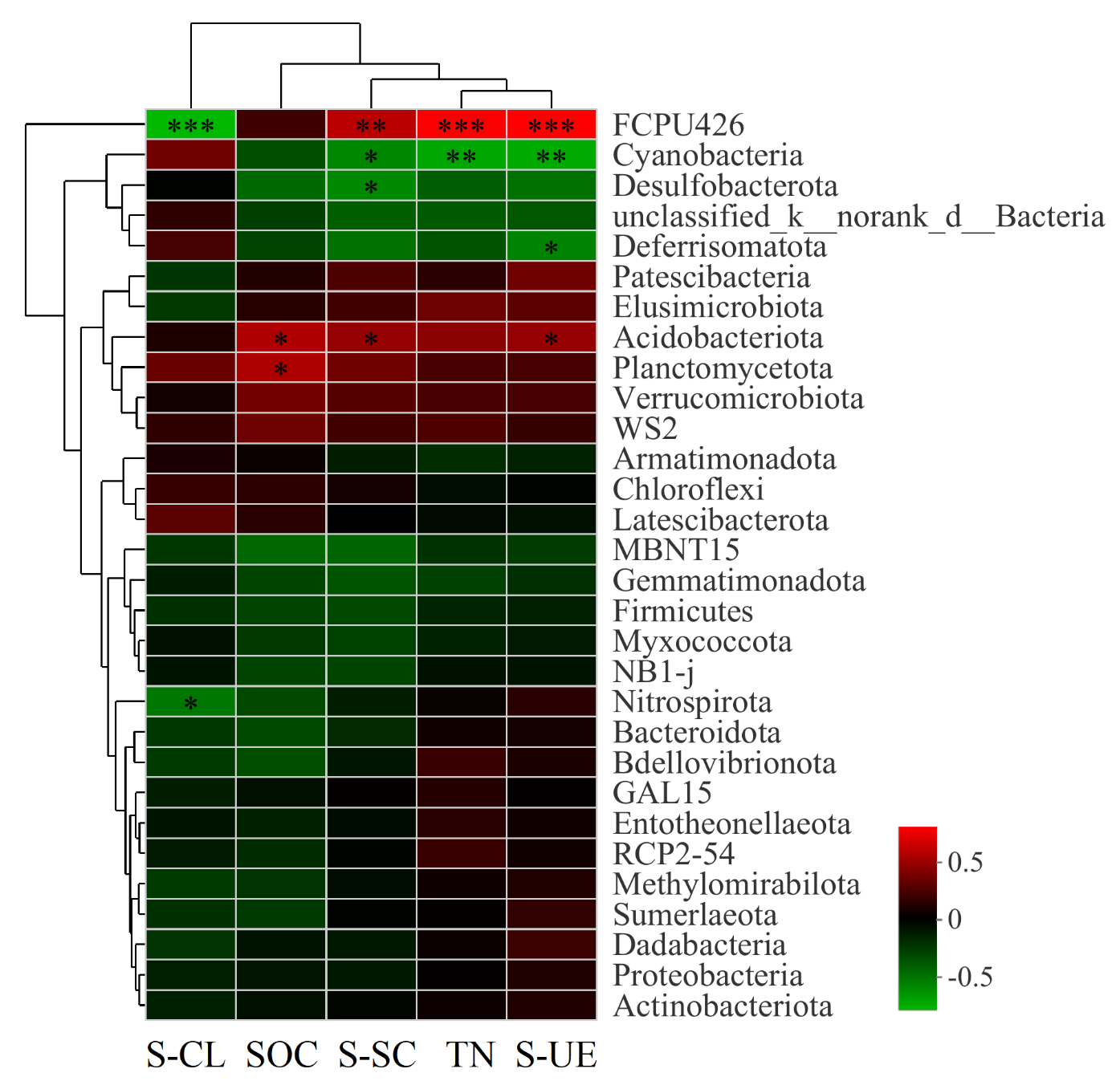


**Figure S7.** Spearman correlation of soil bacterial during spring (A) and in autumn (B) seasons.

Abbreviations: SR-N0 = straw returning without N fertilizer application; SR-N200 = straw returning with 200 kg ha^-1^ N fertilizer application; TP-N0 = traditional planting without N fertilizer application; TP-N200 = traditional planting with 200 kg/ha nitrogen fertilizer application.


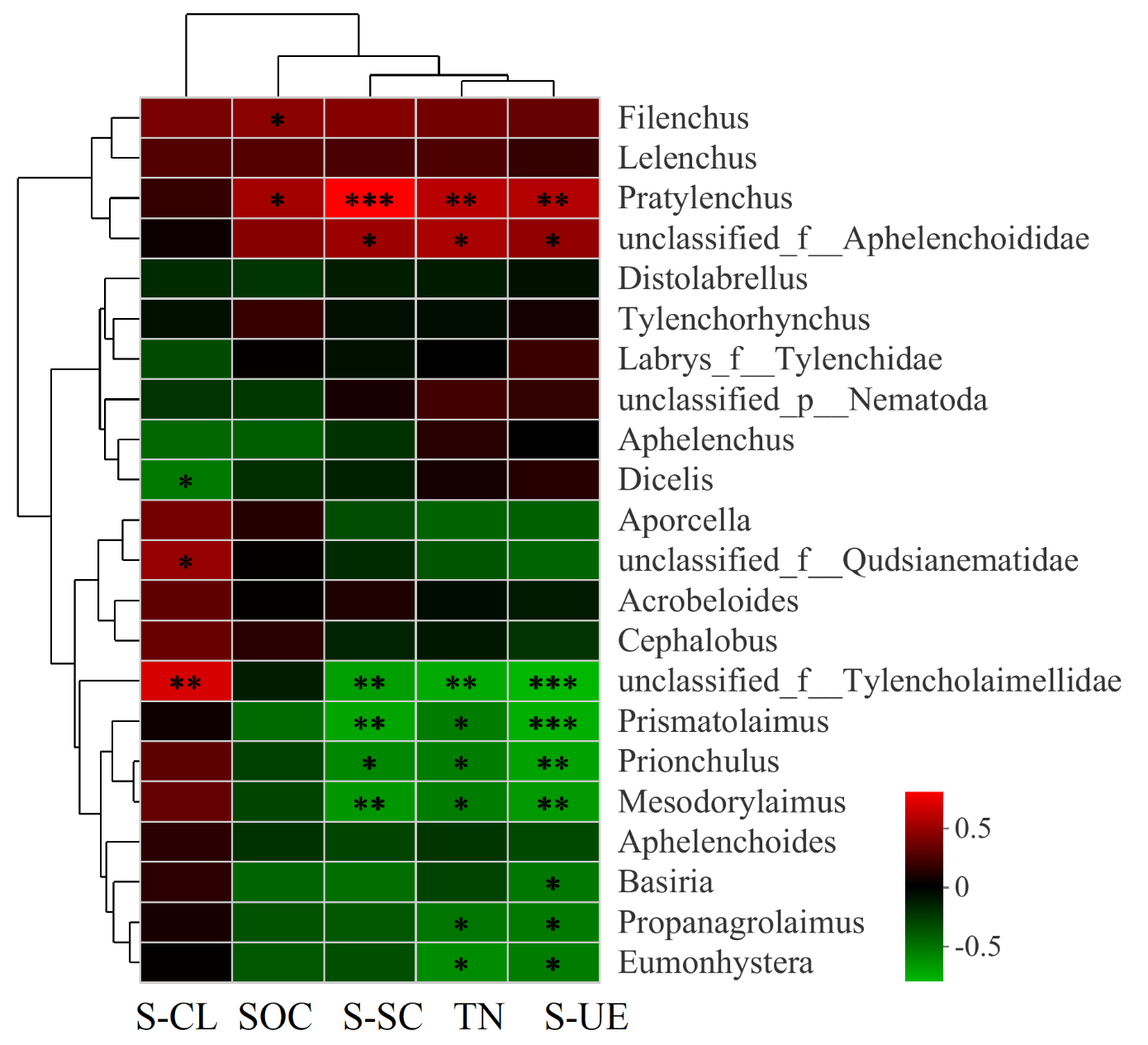


**Figure S8.** Spearman correlation of soil fungi during spring (A) and in autumn (B) seasons.

Abbreviations: SR-N0 = straw returning without N fertilizer application; SR-N200 = straw returning with 200 kg ha^-1^ N fertilizer application; TP-N0 = traditional planting without N fertilizer application; TP-N200 = traditional planting with 200 kg/ha nitrogen fertilizer application.


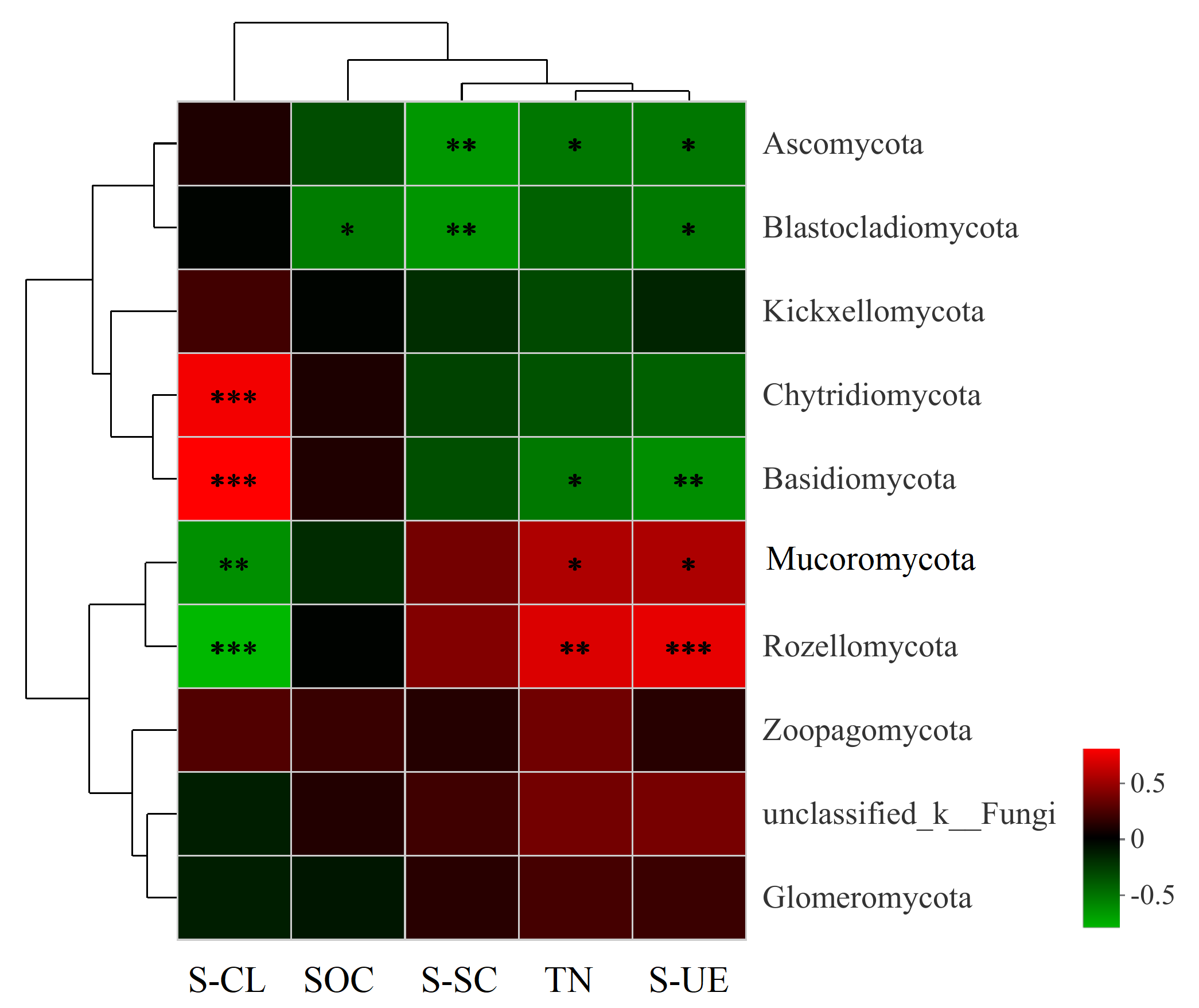


**Figure S9.** Spearman correlation of soil nematode during spring (A) and in autumn (B) seasons.

Abbreviations: SR-N0 = straw returning without N fertilizer application; SR-N200 = straw returning with 200 kg ha^-1^ N fertilizer application; TP-N0 = traditional planting without N fertilizer application; TP-N200 = traditional planting with 200 kg/ha nitrogen fertilizer application.
